# Mpox Awareness, Risk Reduction, and Vaccine Acceptance among People with HIV in Washington, DC

**DOI:** 10.3390/pathogens13020124

**Published:** 2024-01-28

**Authors:** Elisabeth W. Andersen, Paige Kulie, Amanda D. Castel, Jose Lucar, Debra Benator, Alan E. Greenberg, Anne Monroe

**Affiliations:** 1 Department of Epidemiology, Milken Institute School of Public Health, The George Washington University, Washington, DC 20052, USA; elisabeth.andersen@gwu.edu (E.W.A.); pkulie2@gwu.edu (P.K.); aeg1@gwu.edu (A.E.G.); amonroe@gwu.edu (A.M.); 2 Division of Infectious Diseases, The George Washington School of Medicine and Health Sciences, Washington, DC 20052, USA; 3 The Washington DC Veterans Affairs Administration, Washington, DC 20422, USA; debra.benator@va.gov

**Keywords:** mpox, vaccination, risk reduction, awareness, behavior change, knowledge, HIV

## Abstract

People with HIV (PWH) are disproportionally affected by mpox and at risk of severe complications. We assessed mpox knowledge, adoption of preventive behaviors, and vaccination attitudes among PWH enrolled in a longitudinal HIV cohort in Washington, DC, the DC Cohort. We conducted uni- and multivariable analyses comparing participants by vaccination status and HIV risk group, and multinomial regression to identify factors associated with vaccine acceptance. Among 430 PWH, 378 (87.9%) were aware of mpox. Among 373 participants with vaccination status data, 101 (27.1%) were vaccinated, 129 (34.6%) planned to vaccinate, and 143 (38.3%) did not plan to vaccinate. The three vaccination groups differed significantly by age, race, education, HIV risk group, recent STI status, and level of mpox worry (all *p* < 0.05). A higher proportion of men who have sex with men (MSM) reported limiting their number of sexual partners compared to non-MSM (*p* < 0.0001). Multinomial regression models comparing vaccinated to unvaccinated PWH found age, education, mode of HIV transmission/gender, and survey period were significantly associated with vaccination status (all *p* < 0.05). High levels of mpox awareness were observed among this cohort of PWH with more MSM employing risk reduction behaviors and being vaccinated. Ensuring that PWH, regardless of gender, sexual orientation, or age, understand the risks of mpox may improve vaccination uptake.

## 1. Introduction

In the midst of the COVID-19 pandemic, the World Health Organization declared mpox (formerly monkeypox) a public health emergency in July 2022 [1]. Soon thereafter, on 4 August 2022, the United States (US) declared a public health emergency due to the identification of mpox outbreaks occurring across multiple states [2,3]. As of September 2023, there have been more than 90,000 reported cases of mpox globally [4]. Based on the demographics of the outbreak, the US Centers for Disease Control and Prevention (CDC) identified specific groups of people for prevention and vaccination including “gay, bisexual, and other men who have sex with men, gender diverse persons, racial and ethnic minorities, and those who are immunocompromised including people with HIV (PWH)” [5,6].

PWH are not only disproportionally affected by mpox but are also at higher risk of severe complications compared to people without HIV, particularly those with low CD4 counts [7,8]. In the US, as many as 57% of mpox cases have occurred among PWH [6]; and amongst cases of mpox requiring hospitalization as many as 82% were PWH [9]. Among PWH with severe manifestations of mpox, 90% were receiving ART prior to diagnosis, 93% had a CD4 count less than or equal to 200 copies/mm^3^, 28% had a co-occurring sexually transmitted infection (STI), and 23% were unstably housed [9]. In an analysis which matched mpox, HIV, and STI surveillance data across eight jurisdictions, HIV prevalence was 38%, and 41% of patients had at least one documented STI in the prior year [8]. Among persons with HIV and mpox coinfection (*n* = 755), the median interval since HIV diagnosis was 10 years, 94% had received some form of HIV care in the prior year, 82% had a viral load of <200 copies/mL, and 78% had a CD4 ≥ 350 cells/mm^3^. With respect to disease severity, a higher proportion of PWH required hospitalization compared to those without HIV (8% vs. 3%) [8].

Currently, CDC guidance includes immunization with the JYNNEOS vaccine (live, attenuated orthopoxvirus-based) for both pre- and post-exposure prophylaxis in individuals at higher risk of mpox infection, including PWH [10,11]. Additionally, behavioral strategies have been a mainstay of prevention including the use of harm reduction and preventive behavioral measures such as avoiding enclosed spaces such as clubs or parties that might result in skin-to-skin contact with others [4]. Much of the published literature to date on behavioral changes, perceived risk, and vaccine uptake related to mpox has focused on gay, bisexual, and other men who have sex with men (MSM). In the US, a study of MSM from the American Men’s Internet Survey (AMIS), found that approximately half of AMIS participants had adopted some risk reduction strategies in response to the mpox outbreak [12].

Given that mpox vaccines have been proven to be protective, monitoring vaccine hesitancy has also been integral to the outbreak response. In a study among MSM living with HIV in France, among 102 participants, 45% felt at risk of mpox infection; 60% reported wanting to get vaccinated; 40% were hesitant; and 98% were vaccinated against SARS-CoV-2 [13]. Among those who felt at risk, 7.5% were vaccine hesitant and 69% were vaccine accepting [13]. Similarly, a study among 722 MSM living with HIV in China who reported anal sex in the previous year also found relatively high mpox vaccine acceptance with 92% of respondents willing to be vaccinated [14].

In Washington, DC, a city with a high incidence of HIV, the first case of confirmed mpox in a DC resident was reported on 4 June 2022 [15]. As of September 2023, there have been 543 cases reported in DC, of which 96% were among males, 40% were among non-Hispanic Black individuals and 61% were among individuals who self-reported being gay or bisexual [15]. Public health officials in Washington, DC launched an aggressive outbreak response strategy, which included vaccination of over 41,000 persons. Vaccine eligibility included all people regardless of sexual orientation or gender who had multiple sexual partners in the prior two weeks, men who have sex with men who are not in monogamous partnerships, sex workers and staff of venues where sexual activity occurs, and people who are living with HIV or had a sexually transmitted infection diagnosed in the prior three months [16]. Mpox vaccination was widely available at walk-in sites, pop-up clinics, and community-based clinics. Aggressive contact tracing and testing, and access to tecovirimat for treatment of severe mpox were also instituted as part of the DC outbreak response [17].

While several studies have documented mpox knowledge and attitudes among MSM and others behaviorally at risk of mpox, few have focused primarily on vaccine uptake and behavior change specifically among PWH. Moreover, to our knowledge, none have provided data on women living with HIV nor have they been able to provide data on a large sample of Black PWH. Given the high rates of HIV in DC, the risk factors associated with mpox infection among PWH, and the broad response instituted by DC Health inclusive of vaccination among all PWH, this study sought to assess knowledge and perceptions of mpox, attitudes towards vaccination, and adoption of preventive behaviors among a large racial/ethnic and gender diverse cohort of people living with HIV.

## 2. Materials and Methods

### 2.1. Study Participants

Data for this study are from the DC Cohort, a longitudinal prospective cohort study of people with HIV receiving outpatient HIV care in Washington, DC at 14 participating clinical sites. Details of the DC Cohort methods have been described previously [18]. After providing informed consent, upon enrollment into the Cohort, a participant’s baseline data (e.g., HIV risk group, current and nadir CD4, current viral load, history of opportunistic infections, comorbidities, and HIV diagnosis date amongst other variables) are abstracted from the participant’s electronic medical record by a Research Assistant. After enrollment, monthly data exports from participating sites provide updated information about a participant’s HIV and other related medical care. The DC Cohort has been continuously enrolling patients since January 2011 with over 12,000 people enrolled since the study start.

### 2.2. Mpox Survey Measures

DC Cohort participants were recruited to complete a cross-sectional online survey on questions regarding mpox from August 2022 to January 2023. This survey was originally launched during the COVID-19 pandemic and was modified to include questions related to mpox in August 2022. Participants were eligible for this survey if they were (1) an active DC Cohort participant (active participants are those who have had a CD4 or viral load lab or an HIV care visit in the last 18 months), (2) English-speaking, (3) 18 years of age and older, and (4) able to provide informed consent.

Eligible participants were contacted by clinic Research Assistants via phone, email, or during a regular clinic visit and asked to participate in the survey. Participants who agreed were either provided with a REDCap link to complete the survey electronically or were provided with an electronic tablet to complete the survey during a clinic visit [19]. Participants were remunerated with a $25 gift card.

The mpox survey questions were adapted from several ongoing mpox surveys [20,21]. The mpox survey included questions about participants’ awareness of mpox and sources of information (i.e., “Have you heard of monkeypox, mpx, or mpox?”); any exposures or mpox infections they may have had (i.e., “Have you contracted monkeypox?”), as well as applicable treatment (i.e., “Did you receive treatment for monkeypox sometimes called TPOXX or tecovirimat?”); worries about mpox (i.e., “How worried are you about monkeypox?” A lot, a little, not at all); behavioral modifications to prevent infection; and the negative psychosocial impact of the mpox outbreak in the prior month (e.g., impact on one’s emotional state, well-being, intimacy/sex life). Mpox vaccination status and plans to vaccinate were determined based on the response to the question: “Have you received the monkeypox vaccine?” The possible responses were: Yes; No but I plan to; and No, and I do not plan to”. (See Appendix A for the full survey).

### 2.3. Statistical Analysis

Data from the DC Cohort database were linked to the mpox survey results via the DC Cohort participant unique study identifier. Descriptive and bivariable analyses were conducted comparing participants by vaccination status (vaccinated, plan to vaccinate, no plan to vaccinate) and by HIV risk group (MSM, non-MSM males, females). Chi-square tests were used to compare categorical variables and t-tests or ANOVA for continuous variables. Multinomial regression models were fitted to identify factors associated with mpox vaccine acceptance and included the following initial covariates: age, race/ethnicity, education, gender/mode of transmission, and survey period. The effect of age was fitted using a restricted cubic spline with three knots to consider a possible nonlinear effect of age on the probability of vaccination. To simplify the model, given the relatively small sample size, factors were removed in a stepwise fashion and refitted in each step. The final model included age, education, gender/mode of transmission, education, and survey period. *p*-values of less than or equal to 0.05 were considered statistically significant. Regression results were reported as odds ratios and 95% confidence intervals. The final regression model was used to predict the probability of vaccination status for different combinations of the significant factors. All analyses were conducted in SAS (Cary, NC, USA) and R.

## 3. Results

### 3.1. Awareness of Mpox

A total of 430 PWH completed the mpox survey questions, which were then linked to DC Cohort data. Of these, 378 participants (88%) reported being aware of mpox. When comparing those who had heard about mpox to those who had not, statistically significant differences were observed by race (*p* = 0.0261) and by the number of people living in a household (*p* = 0.0002) (Table 1). Awareness did not differ based on age, HIV mode of transmission, education level, or time period of survey completion. Among the 378 participants who had previously heard of mpox, a majority of them (60.7%) learned about mpox from online, TV, or print news. Of these 378, 12 (3.2%) had previously contracted mpox.

Those PWH who reported having mpox were of a mean age of 36.8 (SD7.84), 92% were male, 75% were non-Hispanic Black, and 75% were single. The median time since HIV diagnosis was 12 years, and five (45.5%) were on antiretroviral therapy at the time of diagnosis. Clinically, the most recent median viral load was 30 copies/mL, and the most recent median CD4 count was 619 cells/µL (66.7%). Eight (66.7%) participants had an HIV care visit within 12 months of survey completion, and one person had an STI diagnosis in the 6 months prior to survey completion. Nine of the 12 (75%) participants had received at least one dose of the mpox vaccine, and 50% received tecovirimat under the CDC’s expanded access protocol.

### 3.2. Vaccination Status

Vaccination status information was unavailable for five participants, leaving 373 participants with data on vaccination status. Table 2 describes the demographic and HIV clinical factors of participants by vaccination status. Overall, 101 (27.1%) participants were vaccinated, 129 (34.6%) had not yet been vaccinated but planned to, and 143 (38.3%) participants did not plan to get an mpox vaccination. Among the vaccinated, 43.6% were vaccinated at a health department clinic, 42.6% were vaccinated at a community health clinic, and 10.9% were vaccinated at their healthcare provider’s office. Four percent of respondents reported their reason for vaccination was due to a known exposure.

Those who were vaccinated were significantly younger than the unvaccinated (median 46 years vs. 57 years (plan to vaccinate) vs. 56 years (no plans to vaccinate) *p* < 0.0001) and included a higher proportion of males (96% vs. 74.4% vs. 57.7%, *p* < 0.0001). A higher proportion of non-Blacks were vaccinated (33% vs. 16.7% vs. 15.2%, *p* = 0.0015) and those who were vaccinated included a higher proportion of college graduates (61.4% vs. 25.8% vs. 30.3%, *p* < 0.0001). The highest proportion of vaccinated participants were MSM (73% vs. 43.2% vs. 27.5%, *p* < 0.0001). Participants who decided not to be vaccinated included a higher proportion of not being worried about mpox at all (60.6%) compared to the vaccinated group (33.0%) and planning to vaccinate group (21.1%) (*p* < 0.0001). A higher proportion of participants who completed the survey in the later time period were vaccinated (61.4% vs. 40.3% vs. 53.1%, *p* = 0.0052).

Clinically, those who were vaccinated had been living with HIV for a significantly shorter time period than the unvaccinated (median 15.6 years vs. 19.8 years vs. 20.3 years *p* = 0.0019). A higher proportion of vaccinated participants had an STI diagnosed in the past 18 months compared to both unvaccinated groups (18.3% vs. 6.6% and 3.5%, *p* = 0.0009). Additionally, a higher proportion of participants in the vaccinated group had CD4 values above 500 cells/mm^3^ (82% vs. 72.6% and 59.4%, *p* = 0.0007). Participants vaccinated for mpox and those who planned to vaccinate were also statistically significantly more likely to be vaccinated for COVID-19 (95% vs. 95.3% vs. 88%, *p* = 0.0383) and to have fewer comorbidities. There were no statistically significant differences found between vaccination groups with respect to relationship status, STI screening in the prior 18 months, receipt of an influenza vaccine, currently being on ART, or viral suppression.

### 3.3. Behavior Change and Vaccination Status

Table 3 shows several significant associations between vaccination group and behavior change in relation to mpox. Among those who were vaccinated, 34.7% of participants limited their number of sexual partners compared to 7% of those participants who did not intend to be vaccinated (*p* < 0.0001). Among those who were vaccinated, 14.9% of participants were only having sex with a certain group of people or not meeting new partners in comparison to 6.3% of the group who did not plan to be vaccinated (*p* = 0.04). Additionally, 9.9% of vaccinated participants talked to their partners about mpox symptoms before sex compared to 2.8% of those with no plan to vaccinate (*p* = 0.03). There were no statistical differences in canceling travel plans, limiting social gatherings, avoiding skin-to-skin contact, or condom use by vaccination status.

### 3.4. Behavior Change and Negative Impact of Mpox by Mode of HIV Transmission

**Figure 1:**
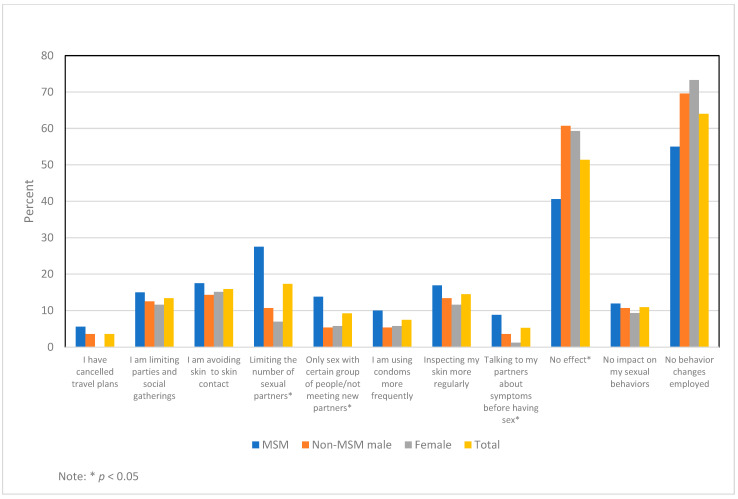
Mpox Behavior Change by Mode of HIV Transmission.

Given the public health emphasis placed on limiting transmission among MSM, we sought to determine if behavior change varied by mode of HIV transmission. When comparing MSM to non-MSM males and females, we found that higher proportions of MSM were limiting their numbers of sexual partners (*p* < 0.0001), were only having sex with a certain group of people or not meeting new partners (*p* = 0.0285), were talking to their partners about mpox symptoms before having sex, (*p* = 0.0249), and reported more effects on their overall behavior (*p* = 0.0012) (Figure 1). We also assessed the negative psychosocial impacts of mpox by mode of HIV transmission group. The only factor found to be statistically significantly different between transmission groups was intimacy/sex, with 20% of MSM indicating a negative impact on intimacy/sex compared to 6.2% of non-MSM males and 3.5% of females (Supplemental Table S1).

### 3.5. Factors Associated with Vaccination Status

**Figure 2:**
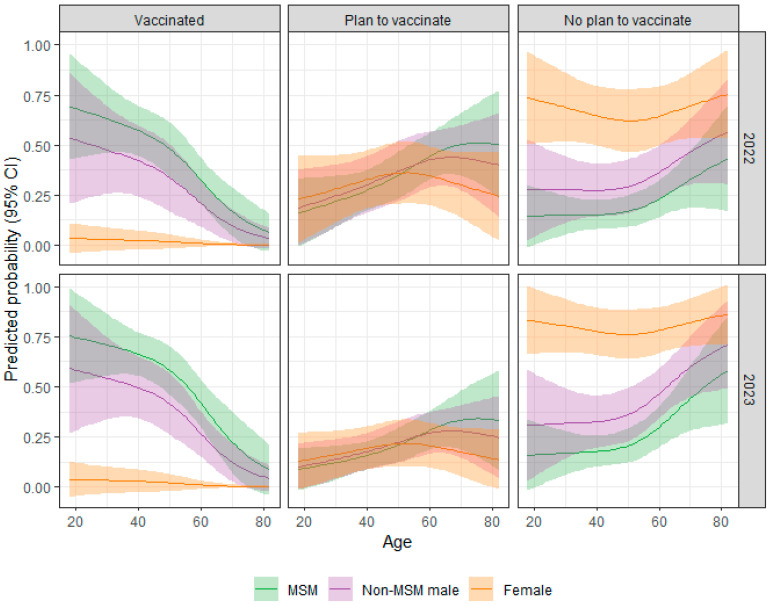
Predicted Probability of Vaccine Group (with 95% CI) by Age, HIV Mode of Transmission/Gender, Survey Year, and College Education.

A multinomial logistic regression model of the association of selected clinical factors including CD4 count, viral suppression (i.e., HIV RNA < 200 copies/mL), being on antiretroviral therapy, and co-morbidities on vaccination group was conducted. There were no significant associations identified (See Supplemental Tables S2 and S3). A second multinomial logistic regression model of the association of demographic variables and year of survey administration on the three vaccine groups with the “do not plan to vaccinate” group as the reference group was conducted. To simplify the model, factors were removed in a stepwise fashion and refitted in each step. Race was removed as it had the highest *p*-value at 0.1185. Younger PWH, those with higher educational attainment, and MSM were more likely to vaccinate compared to those not planning on vaccinating. The final model included age (overall *p* = 0.0003), education (*p* = 0.0496), mode of HIV transmission and gender (overall *p* < 0.0001), and survey year (overall *p* = 0.0134) (Table 4). The predicted probability of the three vaccination groups by mode of transmission, gender, age, survey year, and college education are seen in Figure 2. College-educated females had a high probability of belonging to the “do not plan to vaccinate” group for all ages and in both survey periods whereas MSM and non-MSM males had a higher probability of belonging to the “vaccinate” group, but the probability decreased with age.

## 4. Discussion

In this analysis of an mpox survey completed by participants of a longitudinal HIV cohort, we found a relatively high level of mpox awareness with 88% of PWH reporting being aware of mpox. This level of knowledge is within the range of other studies of sexual minority men in China (37%) and Brazil (97%) [14,22]. Additionally, more than a quarter of PWH (27%) received the mpox vaccine with an additional 35% planning on getting vaccinated. We also found that vaccinated participants were more likely to be MSM, had higher educational attainment, were non-Black, and were more worried about mpox. Vaccinated PWH had higher CD4 values, were more recently diagnosed with HIV, had an STI diagnosed in the past 18 months, and had received a COVID-19 vaccine.

With more than 60% of participants receiving the vaccine or planning to get vaccinated, our findings are on the lower end of other studies that also reported mpox vaccine uptake (or willingness to vaccinate) ranging from 51% to 95% among at-risk populations [10,23,24,25,26,27]. In a study of transgender people and gay and bisexual men who have sex with men (T/GBM) from an STI clinic in British Columbia, 66% of participants identifying as T/GBM had been vaccinated, and being unvaccinated was more common in participants who identified as bisexual or heteroflexible/mostly straight [26]. Additionally, an online-based cross-sectional study of a convenience sample of adults in Brazil in which 96.7% of participants were gay, bisexual, or pansexual found that 95.1% were willing to be vaccinated for mpox [22]. In a convenience sample of MSM from the American Men’s Internet Survey (AMIS) study, almost one in five (18.6%) respondents reported receiving at least one dose of the JYNNEOS vaccine with 22.3% of PWH reporting receiving at least one vaccine dose [12].

Given the historical nature of HIV and its devastating impact on the LGBTQ community, coupled with the fact that the global mpox outbreak was initially largely concentrated in the MSM population, it is not surprising that we observed higher rates of not only worry but vaccination among this group of individuals. Other published studies corroborate our findings that MSM both with and without HIV with higher self-perceived risk and those more worried about contracting mpox were more likely to accept vaccination [10,13,24]. Participants who were vaccinated or planned to vaccinate may also have been at higher risk for mpox given their recent histories of STIs. Nevertheless, they were also more engaged in preventive health measures such as having been vaccinated for COVID-19 and having higher CD4 counts. In the U.S., more than half (53%) of AMIS participants reported being somewhat or very concerned about mpox, yet 82% felt confident they could protect themselves from infection [12]. Similarly, a study in the Netherlands found MSM living with HIV were significantly more likely to perceive themselves as having a high or very high risk of acquiring mpox infection compared to MSM without HIV [28]. Among those surveyed, 68% expressed some concern and 64% perceived their risk to be high or very high. In a study in China among MSM, vaccination willingness was associated with a higher level of knowledge of preventive measures, higher perceived susceptibility to infection, and lower consistency of condom use. Interestingly, in that analysis, higher vaccination willingness was associated with a lower educational level [14].

In DC, specifically, the low barrier approach to vaccination, inclusive of many risk groups including all PWH, may also help explain the high observed vaccination rates and intent to vaccinate among our respondents. The fact that DC Health issued Health Notices to clinical providers regarding the outbreak and provided clinical recommendations, which included free vaccination, and offered expanded vaccine eligibility for both PWH and persons of any sexual orientation or gender likely fostered high vaccine uptake [15]. Almost one quarter (22.7%) of female survey participants were vaccinated or planned to vaccinate against mpox, illustrating the broader impact of the DC-specific vaccination approach in protecting women living with HIV as well.

With respect to adopting protective behaviors for mpox prevention, in our survey, PWH who were vaccinated and MSM were more likely to limit their number of sexual partners, only have sex with certain types of partners, and discuss mpox with their partners before sex than other males and females. These reported changes in intimacy and sexual behaviors found in our study population have also been observed in other studies. A study of participants recruited from sexual health clinics in Victoria, Australia found that half of their participants reported that they would reduce having sex with casual partners, stop having sex with concurrent drug use, stop attending sex-on-premises venues, and stop having group sex [27]. They also found that a quarter of their participants indicated they would increase condom use [27]. Similarly, the AMIS study found that almost half of their MSM participants were employing risk reduction behaviors. Forty-eight percent of AMIS respondents reported reducing their number of sexual partners, and half reported limiting the number of one-time sexual encounters and the number of partners they met online or through dating apps and sex venues [12]. Additionally, 42% of AMIS participants reduced attendance at sex venues or social events with the potential for close contact [12]. Employing these behavior changes, in concert with vaccination, likely helped limit the spread of mpox among PWH in DC and globally.

While overall knowledge and vaccine willingness were high, there were still disparities observed with respect to education and race, with Blacks and those of lower educational attainment less likely to seek vaccination. These findings were also observed among PWH in DC regarding COVID-19 vaccinations and emphasize the need for equitable public health education and strategies that reach everyone who will benefit [29]. Models of culturally competent community education and mobilization efforts were effective in enhancing mpox vaccine distribution in DC. DC Health partnered with community-based organizations such as Us Helping Us and Whitman Walker Health, organizations that are knowledgeable about and highly experienced in providing services for sexual and racial/minority groups, which in turn helped increase rates of mpox awareness and vaccination among minority MSM [17,30]. These models could be adapted to engage other communities affected by or at-risk of mpox to improve knowledge, vaccine acceptability, and vaccine uptake for mpox, COVID-19, and other emerging infectious diseases [31].

There were several limitations to this study. First, it was conducted among a cohort of PWH who are receiving HIV care in Washington, DC and enrolled in the DC Cohort. Cohort participants are enrolled in the overall study at the time of a clinical visit at a participating site and were contacted either in-person, via telephone or via email to complete this survey which may bias our sample. The self-reported nature of the survey, which asked about behavior change, may also have resulted in social desirability bias. Additionally, we were unable to determine whether providers were differentially recommending mpox vaccination nor do we have data on healthcare access, though Cohort participants are likely more engaged in care [32]. The majority of Cohort participants are Black, and above the age of 50, which mirrors the DC HIV epidemic; however, these results may not be generalizable to other populations or locations [32,33]. Eligible survey participants needed to have been seen for an HIV care visit within the last 18 months, so these results may also not hold true for PWH who are not currently in care. Further, the survey was only available in English, thus it is not reflective of PWH enrolled in the DC Cohort whose primary language is not English. Additionally, the cross-sectional nature of the survey precluded us from determining whether those who planned to vaccinate actually received the mpox vaccination. Finally, while we based vaccination coverage on self-reporting, we are currently conducting a review of vaccination information at DC Cohort clinical sites to estimate the true rate of mpox vaccination coverage among this cohort of PWH. Nevertheless, this analysis provides data on one of the largest studies of PWH in the US and vaccine uptake and behavior changes as it relates to mpox. It also provides data on a primarily Black and urban population of PWH.

## 5. Conclusions

Our analysis suggests that health education about the risks of mpox and preventive measures led to the successful adoption of risk reduction behaviors among PWH in DC. Furthermore, the city’s response to the outbreak serves as a model for lowering barriers to vaccine eligibility, access, and receipt, all of which were paramount to curtailing the outbreak locally. As infectious diseases such as mpox and SARS-CoV-2 continue to emerge, assessing the impact of these viruses on PWH and developing timely and effective strategies to protect PWH will be necessary. Specifically, raising awareness in all PWH, regardless of gender and age, about the risks of mpox and encouraging vaccination with particular emphasis on those who have recently been diagnosed with STIs is warranted. Moreover, when strategies such as behavioral modification and delivery of novel vaccines are employed, we must be ready to educate PWH about these strategies and emphasize both the risks and benefits of employing them to ensure optimal public health outcomes and disease control.

## Figures and Tables

**Table 1:** Awareness of Mpox among DC Cohort Participants, *N* = 430.

	Mpox Awareness			
Characteristic	Yes *N* = 378	No *N* = 52	Total *N* = 430	*p*-Value
**Age (median, IQR)**	54.0 [43.0;62.0]	52.5 [41.0;60.0]	54.0 [43.0;62.0]	0.3963
Gender *				0.8443
Male	278 (73.7%)	39 (75.0%)	317 (73.9%)	
Female	91 (24.1%)	13 (25.0%)	104 (24.2%)	
Other	8 (2.1%)	0 (0%)	8 (1.9%)	
**Race**				**0.0261**
Black/African American	291 (77.0%)	48 (92.3%)	339 (78.8%)	
White	51 (13.5%)	1 (1.9%)	52 (12.1%)	
More than One Race	17 (4.5%)	1 (1.9%)	18 (4.2%)	
Asian	2 (0.5%)	0 (0%)	2 (0.5%)	
American Indian/Alaska Native	0 (0%)	1 (1.9%)	1 (0.2%)	
Native Hawaiian or Other Pacific Islander	0 (0%)	0 (0%)	0 (0%)	
Other	8 (2.1%)	1 (1.9%)	9 (2.1%)	
Unknown or Not Reported	9 (2.4%)	0 (0%)	9 (2.1%)	
**Hispanic**				0.7127
Yes	15 (4.0%)	3 (5.8%)	18 (4.2%)	
No	350 (92.6%)	48 (92.3%)	398 (92.6%)	
Decline to answer	13 (3.4%)	1 (1.9%)	14 (3.3%)	
Education *				0.5245
Less than a high school diploma	27 (7.2%)	3 (5.9%)	30 (7.0%)	
Grade 12 or GED (High school graduate)	109 (29.0%)	20 (39.2%)	129 (30.2%)	
College 1 year to 3 years (Some college or technical school)	101 (26.9%)	12 (23.5%)	113 (26.5%)	
College 4 years or more (College graduate)	139 (37.0%)	16 (31.4%)	155 (36.3%)	
Mode of HIV transmission **				0.0979
MSM	168 (45.2%)	14 (29.2%)	182 (43.3%)	
Heterosexual	103 (27.7%)	21 (43.8%)	124 (29.5%)	
IDU	20 (5.4%)	2 (4.2%)	22 (5.2%)	
Perinatal	11 (3.0%)	3 (6.2%)	14 (3.3%)	
Other	70 (18.8%)	8 (16.7%)	78 (18.6%)	
Relationship Status *				0.1287
Single, never married	204 (55.4%)	29 (55.8%)	233 (55.5%)	
Married or living with partner	77 (20.9%)	11 (21.2%)	88 (21.0%)	
Divorced, separated, or widowed	79 (21.5%)	8 (15.4%)	87 (20.7%)	
Other	8 (2.2%)	4 (7.7%)	12 (2.9%)	
Number of people living in the household *				**0.0002**
1	167 (44.3%)	16 (30.8%)	183 (42.7%)	
2	122 (32.4%)	8 (15.4%)	130 (30.3%)	
3	40 (10.6%)	13 (25.0%)	53 (12.4%)	
4	19 (5.0%)	6 (11.5%)	25 (5.8%)	
5+	29 (7.7%)	9 (17.3%)	38 (8.9%)	
**Annual household income**				0.4465
<$25,000	108 (28.6%)	16 (30.8%)	124 (28.8%)	
$25,000–$34,999	48 (12.7%)	5 (9.6%)	53 (12.3%)	
$35,000–$49,999	38 (10.1%)	5 (9.6%)	43 (10.0%)	
$50,000–$99,999	69 (18.3%)	7 (13.5%)	76 (17.7%)	
$10,000+	66 (17.5%)	7 (13.5%)	73 (17.0%)	
Don’t know/Not sure	22 (5.8%)	7 (13.5%)	29 (6.7%)	
Decline to answer	27 (7.1%)	5 (9.6%)	32 (7.4%)	
**Survey Completion Period**				0.1459
August-December 22	186 (49.2%)	20 (38.5%)	206 (47.9%)	
January-April 23	192 (50.8%)	32 (61.5%)	224 (52.1%)	

* Excludes missing responses: gender identity (*n* = 1); education (*n* = 3); mode of HIV transmission (*n* = 10); relationship status (*n* = 10); number of people living in the household (*n* = 1). ** Mode of transmission was based on DC Cohort data and not self-reported. All other variables were self-reported in the survey. Bolded *p*-values indicate statistical significance.

**Table 2:** Participant Demographic, Behavioral, and HIV Clinical Characteristics by Mpox Vaccination Status, *N* = 373.

	Mpox Vaccination Status				
Characteristic	Yes *N* = 101	No, But Plan to Vaccinate *N* = 129	No Plans to Vaccinate *N* = 143	Total *N* = 373	*p*-Value
**Demographics**					
**Age (median, IQR)**	46.0 [38.0;56.0]	57.0 [47.0;63.0]	56.0 [44.0;63.0]	54.0 [43.0;62.0]	**<0.0001**
Gender *					**<0.0001**
Male	97 (96.0%)	96 (74.4%)	82 (57.7%)	275 (73.9%)	
Female	2 (2.0%)	28 (21.7%)	59 (41.5%)	89 (23.9%)	
Other	2 (2.0%)	5 (3.9%)	1 (0.7%)	8 (2.2%)	
Race *					**0.0015**
Black/African American	67 (67.0%)	105 (83.3%)	117 (84.8%)	289 (79.4%)	
All other	33 (33.0%)	21 (16.7%)	21 (15.2%)	75 (20.6%)	
**Hispanic**					0.1314
Yes	7 (6.9%)	5 (3.9%)	1 (0.7%)	13 (3.5%)	
No	90 (89.1%)	120 (93.0%)	137 (95.8%)	347 (93.0%)	
Decline to answer	4 (4.0%)	4 (3.1%)	5 (3.5%)	13 (3.5%)	
Education *					**<0.0001**
Less than a high school diploma	2 (2.0%)	10 (7.8%)	15 (10.6%)	27 (7.3%)	
Grade 12 or GED (High school graduate)	16 (15.8%)	48 (37.5%)	44 (31.0%)	108 (29.1%)	
College 1 year to 3 years (Some college or technical school)	21 (20.8%)	37 (28.9%)	40 (28.2%)	98 (26.4%)	
College 4 years or more (College graduate)	62 (61.4%)	33 (25.8%)	43 (30.3%)	138 (37.2%)	
Mode of HIV transmission **					**<0.0001**
MSM	73 (73.0%)	54 (43.2%)	39 (27.5%)	166 (45.2%)	
Heterosexual	7 (7.0%)	38 (30.4%)	55 (38.7%)	100 (27.2%)	
IDU	0 (0%)	6 (4.8%)	14 (9.9%)	20 (5.4%)	
Perinatal	0 (0%)	2 (1.6%)	9 (6.3%)	11 (3.0%)	
Other	20 (20.0%)	25 (20.0%)	25 (17.6%)	70 (19.1%)	
Relationship Status *					0.1591
Divorced, separated, or widowed	15 (15.5%)	24 (18.9%)	38 (27.3%)	77 (21.2%)	
Married or living with partner	18 (18.6%)	28 (22.0%)	31 (22.3%)	77 (21.2%)	
Single, never married	61 (62.9%)	74 (58.3%)	66 (47.5%)	201 (55.4%)	
Other	3 (3.1%)	1 (0.8%)	4 (2.9%)	8 (2.2%)	
Number of people living in the household *					**0.0404**
1	53 (52.5%)	55 (43.0%)	56 (39.2%)	164 (44.1%)	
2	36 (35.6%)	39 (30.5%)	45 (31.5%)	120 (32.3%)	
3	5 (5.0%)	16 (12.5%)	19 (13.3%)	40 (10.8%)	
4	6 (5.9%)	5 (3.9%)	8 (5.6%)	19 (5.1%)	
5+	1 (1.0%)	13 (10.2%)	15 (10.5%)	29 (7.8%)	
**Annual household income**					**0.0006**
<$25,000	12 (11.9%)	45 (34.9%)	50 (35.0%)	107 (28.7%)	
$25,000–$34,999	12 (11.9%)	15 (11.6%)	20 (14.0%)	47 (12.6%)	
$35,000–$49,999	10 (9.9%)	17 (13.2%)	10 (7.0%)	37 (9.9%)	
$50,000–$99,999	22 (21.8%)	21 (16.3%)	26 (18.2%)	69 (18.5%)	
$10,000+	31 (30.7%)	13 (10.1%)	21 (14.7%)	65 (17.4%)	
Don’t know/Not sure	5 (5.0%)	10 (7.8%)	7 (4.9%)	22 (5.9%)	
Decline to answer	9 (8.9%)	8 (6.2%)	9 (6.3%)	26 (7.0%)	
**Survey Completion Period**					**0.0052**
August-December 22	39 (38.6%)	77 (59.7%)	67 (46.9%)	183 (49.1%)	
January-April 23	62 (61.4%)	52 (40.3%)	76 (53.1%)	190 (50.9%)	
**Self-Reported Behavioral Parameters**					
Worry about Mpox *					**<0.0001**
A lot	18 (18.0%)	31 (24.2%)	9 (6.3%)	58 (15.7%)	
A little	49 (49.0%)	70 (54.7%)	47 (33.1%)	166 (44.9%)	
Not at all	33 (33.0%)	27 (21.1%)	86 (60.6%)	146 (39.5%)	
Received COVID-19 vaccine *					**0.0383**
Yes	96 (95.0%)	123 (95.3%)	125 (88.0%)	344 (92.5%)	
No	5 (5.0%)	6 (4.7%)	17 (12.0%)	28 (7.5%)	
Received a flu vaccine *					0.3228
Yes	83 (83.8%)	98 (76.6%)	108 (76.6%)	289 (78.5%)	
No	16 (16.2%)	30 (23.4%)	33 (23.4%)	79 (21.5%)	
**HIV and Clinical Parameters**					
Years since HIV diagnosis (median, IQR) *	15.6 [10.3;22.4]	19.8 [13.5;28.4]	20.3 [13.4;27.8]	18.7 [12.7;26.7]	**0.0019**
Chlamydia test in past 18 months *					0.1334
Yes	53 (64.6%)	62 (58.5%)	57 (50.4%)	172 (57.1%)	
No	29 (35.4%)	44 (41.5%)	56 (49.6%)	129 (42.9%)	
Gonorrhea test in past 18 months *					0.0981
Yes	53 (63.9%)	63 (56.8%)	59 (48.8%)	175 (55.6%)	
No	30 (36.1%)	48 (43.2%)	62 (51.2%)	140 (44.4%)	
Syphilis test in past 18 months *					0.3144
Yes	61 (73.5%)	89 (80.2%)	87 (71.9%)	237 (75.2%)	
No	22 (26.5%)	22 (19.8%)	34 (28.1%)	78 (24.8%)	
Any STI in the past 18 months *					**0.0009**
Yes	15 (18.3%)	7 (6.6%)	4 (3.5%)	26 (8.6%)	
No	67 (81.7%)	99 (93.4%)	109 (96.5%)	275 (91.4%)	
Currently on ART *					0.6269
Yes	80 (84.2%)	95 (79.2%)	106 (82.2%)	281 (81.7%)	
No	15 (15.8%)	25 (20.8%)	23 (17.8%)	63 (18.3%)	
Latest CD4 count >500 cells/mm^3^ *					**0.0007**
Yes	82 (82.0%)	90 (72.6%)	82 (59.4%)	254 (70.2%)	
No	18 (18.0%)	34 (27.4%)	56 (40.6%)	108 (29.8%)	
Last VL suppressed (VL < 200 copies/mL *					0.1008
Yes	80 (92.0%)	112 (94.9%)	117 (87.3%)	309 (91.2%)	
No	7 (8.0%)	6 (5.1%)	17 (12.7%)	30 (8.8%)	
Charlson Score (QCCI)					
0	63 (62.4%)	46 (36.2%)	60 (42.0%)	169 (45.6%)	**<0.001**
1	15 (14.9%)	37 (29.1%)	32 (22.4%)	84 (22.6%)	
2+	23 (22.8%)	44 (34.6%)	51 (35.7%)	118 (31.8%)	
Missing	0	2	0	2	

* Excludes missing responses: gender identity (*n* = 1); race (*n* = 9); relationship status (*n* = 10); education (*n* = 2); number of people in household (*n*- = 1); mode of HIV transmission (*n* = 6); worry about mpox (*n* = 3); chlamydia testing (*n* = 72); gonorrhea testing (*n* = 58); syphilis testing (*n* = 58); any STI in past 18 months (*n* = 72); received COVID vaccine (*n* = 1); received flu vaccine (*n* = 5); current ART (*n* = 29); latest CD4 count and CD4 above 500 cells/mm^3^ (*n* = 11); latest VL suppressed (*n* = 34); years since HIV diagnosis (*n* = 7). ** Mode of transmission, HIV, and other clinical parameters are based on DC Cohort data and not self-reported. All other variables were self-reported in the survey.

**Table 3:** Behavior Change by Vaccination Status.

		MPOX Vaccination Group			Total	*p*-Value
		Yes	No, But I Plan to	No, and I Don’t Plan to		
I have canceled travel plans		4 (4.0%)	6 (4.7%)	4 (2.8%)	14 (3.8%)	0.7183
I am limiting parties and social gatherings		17 (16.8%)	19 (14.7%)	13 (9.1%)	49 (13.1%)	0.1698
I am avoiding skin-to-skin contact		19 (18.8%)	22 (17.1%)	18 (12.6%)	59 (15.8%)	0.3774
Limiting the number of sexual partners		35 (34.7%)	17 (13.2%)	10 (7.0%)	62 (16.6%)	**<0.0001**
Only sex with a certain group of people or not meeting new partners		15 (14.9%)	9 (7.0%)	9 (6.3%)	33 (8.8%)	**0.0444**
I am using condoms more frequently		9 (8.9%)	9 (7.0%)	9 (6.3%)	27 (7.2%)	0.7320
Inspecting my skin more regularly		21 (20.8%)	18 (14.0%)	14 (9.8%)	53 (14.2%)	0.0527
Talking to my partners about symptoms before having sex		10 (9.9%)	5 (3.9%)	4 (2.8%)	19 (5.1%)	**0.0336**
No effect		46 (45.5%)	55 (42.6%)	90 (62.9%)	191 (51.2%)	**0.0015**
No impact on my sexual behaviors		6 (5.9%)	15 (11.6%)	19 (13.3%)	40 (10.7%)	0.1733
Number of behavior changes employed	0	54 (53.5%)	73 (56.6%)	111 (77.6%)	238 (63.8%)	**0.0002**
	1	16 (15.8%)	31 (24.0%)	13 (9.1%)	60 (16.1%)	
	2	11 (10.9%)	8 (6.2%)	8 (5.6%)	27 (7.2%)	
	3+	20 (19.8%)	17 (13.2%)	11 (7.7%)	48 (12.9%)	

**Table 4:** Multinomial Logistic Regression Models for the Three Vaccination Groups (*N*=356) Adjusted for Age Using a Restricted Cubic Spline.

	Vaccine vs. No Vaccine			Plan Vaccine vs. No Vaccine			
	RRR	95% CI	*p*-Value	RRR	95% CI	*p*-Value	*p* All
**Age**							
**Age1**	0.99	0.94–1.05	**0.0002**	1.02	0.98–1.07	0.5650	**0.0003**
**Age2**	0.95	0.89–1.01		0.98	0.93–1.02		
**Education**							
Less than high school	referent		0.1753	referent		0.2455	**0.0496**
High school graduate	2.96	0.32–27.44		1.51	0.58–3.96		
At least some college	5.00	0.57–43.77		0.93	0.36–2.42		
**Mode/gender**							
Non-MSM male	referent		**<0.0001**	referent			**<0.0001**
MSM	2.45	1.23–4.88		1.64	0.86–3.12	**0.0011**	
Female	0.02	0.00–0.19		0.46	0.24–0.89		
**Survey Year**							
2023	referent		0.9869	referent		0.0088	**0.0134**
2022	1.01	0.53–1.89		2.03	1.19–3.45		

RRR: relative risk ratio, 95% confidence interval, *p*-value testing the effect at each level, *p* all: overall effect, Age1 and Age2 are the estimated non-linear age effects.

## Data Availability

Data are not publicly available due to the sensitive nature of the data being collected. Interested investigators can complete a data request form as per DC Cohort Executive Committee policies, which can be submitted to Amanda Castel, the DC Cohort Study Principal Investigator for Executive Committee review and approval. Detailed procedures can be found at https://publichealth.gwu.edu/ accessed 20 January 2024.

## Supplementary Materials

The following supporting information can be downloaded at https://www.mdpi.com/article/10.3390/pathogens13020124/s1: Table S1: HIV Mode of Transmission and Negative Impacts from Mpox; Table S2: Multinomial Logistic Regression Models for Vaccination Group for Selected Clinical Variables. Table S3: Joint Multinomial Logistic Regression Models by Vaccination Status for Selected Clinical Variables.

## Appendix A. DC Cohort Mpox Survey

### Have you heard of monkeypox, mpx, or mpox?

Yes [continue to next question]

No [stop]

### Where have you heard about monkeypox? (select all)

Social media

Online, TV, or print news

Health department

Healthcare provider/doctor/nurse

Family

Friends

Other [please specify]

### Have you received the monkeypox vaccine?

Yes

No, but I plan to

No, and I do not plan to

### [If “No but I plan to”] Did you try to get it?

Yes

No

### Please indicate where you received the vaccine (if you have had more than one shot, you can check all that apply)

My doctor’s office

A community health clinic

A pharmacy

A Department of Health clinic

A mobile delivery van

I can’t remember

Other (please specify)________________

### [If yes to receiving vaccine] Some people get the monkeypox vaccine because they know they were exposed to another person with monkeypox. Others get the vaccine before being exposed. Which applies to you?

I got the vaccine because was exposed to someone with monkeypox

I was not exposed to anyone with monkeypox before I got the vaccine

I’m not sure

### [If “got vaccine because exposed to someone”] How soon after exposure to someone with monkeypox did you get the vaccine?

Within 4 days

Between 5 and 14 days after I was exposed

More than 14 days after I was exposed

I don't remember

### Have you contracted monkeypox?

Yes

No

Not sure

### [If yes to contracted monkeypox] Did you receive treatment for monkeypox (sometimes called TPOXX or tecovirimat)?

Yes

No

Not sure

### How has monkeypox affected your behavior? (select all that apply)

I have canceled travel plans.

I am not attending/limiting my attendance at parties or social gatherings.

I am avoiding skin-to-skin contact.

I am limiting the number of sexual partners that I have.

I am only having sex with a certain group of people or not meeting new partners.

I am using condoms more frequently.

I am visually inspecting my skin more regularly

I am talking to my partners about symptoms before having sex.

Other [please specify]

No effect. [response exclusive to other options]

I don't feel it is having any impact on my sexual behaviors

### How worried are you about monkeypox?

A lot.

A little.

Not at all.

### Over the PAST MONTH, which of the following has applied to you about MPX? (Check all that apply)

I have been exposed

I have a sexual partner or household member who has been diagnosed with it

I have a sexual partner or household member who has symptoms of MPX but has not been tested

I have symptoms

I have tested negative

I have tested positive

I have been hospitalized

I am at risk due to other health conditions

I am at risk due to work exposures

I am at risk due to sexual or intimate exposures

I am worried about my friends, partners, family, or me getting it

None of these

### Over the PAST MONTH, has your life felt NEGATIVELY IMPACTED in any of these ways by MPX? (Check all that apply)

Intimacy/sex

My emotional state

My sense of safety

My sense of well-being

My work

My sense of recovery from the COVID-19 pandemic

My trust in science

My trust in public health

None of these
