# Supplementary material for: Mpox Awareness, Risk Reduction, and Vaccine Acceptance among People with HIV in Washington, DC
Source: Pathogens. 2024 Jan 28;13(2):124. doi: 10.3390/pathogens13020124 (PMC10891655; doi:10.3390/pathogens13020124)
Supplement: Supplementary file 1 [file pathogens-13-00124-s001.zip › Supplemental Table 2 and 3.pdf]

Supplemental Table 2 and 3

Table S2. Multinomial Logistic Regression Models for Vaccination Group for Selected Clinical Variables.[1]

	**Vaccine vs No vaccine**			**Plan vaccine vs No vaccine**			
	**RRR**	**95%CI**	**P**	**RRR**	**95%CI**	**P**	**P all**
**CD4 ≥ 500**							
Yes	1.89	0.93-3.84	0.0772	1.64	0.95-2.84	0.0762	0.1045
No	referent			referent	.		
**VL suppressed**							
Yes	1.27	0.43-3.77	0.6617	2.83	0.98-8.15	0.0545	0.1551
No	referent			referent	.		
**Current ART**							
Yes	1.22	0.52-2.87	0.6429	0.77	0.40-1.50	0.4459	0.4835
No	referent			referent	.		
**QCCI**							
0	referent		0.9430	referent	.	0.2743	0.4337
1	0.97	0.42-2.26		1.71	0.88-3.33		
2+	0.88	0.42-1.83		1.37	0.73-2.55		

1.Each variable was regressed individually adjusting for age and gender/mode of HIV transmission.
QCCI: Quan Charlson Comorbidity Index, RRR: Relative risk ratio, 95% CI: 95% Confidence Interval, P: P-value testing the effect at each level, P all: Overall effect

Table S3. Joint Multinomial Logistic Regression Models by Vaccination Status for Selected Clinical Variables[1]

	Vaccine vs No vaccine			Plan vaccine vs No vaccine			
	**RRR**	**95%CI**	**P**	**RRR**	**95%CI**	**P**	**P all**
**CD4 ≥ 500**							
Yes	1.82	0.83-3.99	0.1355	1.36	0.74-2.48	0.3176	0.2962
No	Referent			Referent			
**VL suppressed**							
Yes	1.16	0.35-3.85	0.8090	3.03	0.92-10.00	0.0694	0.1832
No	Referent			Referent			
**Current ART**							
Yes	0.96	0.39-2.37	0.9326	0.67	0.33-1.35	0.2615	0.4668
No	Referent			Referent			
**QCCI**							
0	Referent		0.9720	Referent		0.0728	0.1620
1	1.09	0.44-2.70		2.35	1.13-4.88		
2+	0.97	0.44-2.17		1.57	0.79-3.13		

1.Adjusted for age and gender/mode of HIV transmission
QCCI: Quan Charlson Comorbidity Index, RRR: Relative risk ratio, 95% CI:95% Confidence Interval, P: P-value testing the effect at each level, P all: Overall effect
