# Supplementary material for: Mpox Awareness, Risk Reduction, and Vaccine Acceptance among People with HIV in Washington, DC
Source: Pathogens. 2024 Jan 28;13(2):124. doi: 10.3390/pathogens13020124 (PMC10891655; doi:10.3390/pathogens13020124)
Supplement: Supplementary file 1 [file pathogens-13-00124-s001.zip › Supplementary Table 1.pdf]

Supplementary Materials

Table S1: HIV Mode of Transmission and Negative Impacts from Mpox

		Risk group			Total	P-Value
		**MSM**	**Non-MSM male**	**Female**		
Intimacy/sex		32 (20.0%)	7 (6.2%)	3 (3.5%)	42 (11.7%)	**0.0001**
My emotional state		12 (7.5%)	10 (8.9%)	6 (7.0%)	28 (7.8%)	0.8614
My sense of safety		21 (13.1%)	11 (9.8%)	8 (9.3%)	40 (11.2%)	0.5702
My sense of well-being		14 (8.8%)	15 (13.4%)	6 (7.0%)	35 (9.8%)	0.2704
My work		7 (4.4%)	5 (4.5%)	3 (3.5%)	15 (4.2%)	0.9324
My sense of recovery from the COVID-19 pandemic		13 (8.1%)	3 (2.7%)	5 (5.8%)	21 (5.9%)	0.1704
My trust in science		5 (3.1%)	4 (3.6%)	6 (7.0%)	15 (4.2%)	0.3291
My trust in public health		11 (6.9%)	9 (8.0%)	8 (9.3%)	28 (7.8%)	0.7916
None of these		108 (67.5%)	84 (75.0%)	70 (81.4%)	262 (73.2%)	0.0557
Cumulative number of reported negative impacts	0	110 (68.8%)	85 (75.9%)	71 (82.6%)	266 (74.3%)	0.1972
	1	22 (13.8%)	13 (11.6%)	6 (7.0%)	41 (11.5%)	
	2+	28 (17.5%)	14 (12.5%)	9 (10.5%)	51 (14.2%)	
